# Brain gray matter abnormalities in progressive supranuclear palsy revisited

**DOI:** 10.18632/oncotarget.20895

**Published:** 2017-09-15

**Authors:** PingLei Pan, Yi Liu, Yang Zhang, Hui Zhao, Xing Ye, Yun Xu

**Affiliations:** ^1^ Department of Neurology, Drum Tower Hospital, Medical School of Nanjing University, Nanjing, PR China; ^2^ Department of Neurology, Affiliated Yancheng Hospital, School of Medicine, Southeast University, Yancheng, PR China; ^3^ The State Key Laboratory of Pharmaceutical Biotechnology, Nanjing University, Nanjing, PR China; ^4^ Jiangsu Key Laboratory for Molecular Medicine, Nanjing University Medical School, Nanjing, PR China; ^5^ Jiangsu Province Stroke Center for Diagnosis and Therapy, Nanjing, PR China; ^6^ Nanjing Neuropsychiatry Clinic Medical Center, Nanjing, PR China

**Keywords:** progressive supranuclear palsy, voxel-based morphometry, meta-analysis, cortical-subcortical circuitries, seed-based d mapping

## Abstract

Whole-brain voxel-based morphometry (VBM) studies of progressive supranuclear palsy (PSP) have demonstrated heterogeneous findings regarding gray matter (GM) abnormalities. Here, we used Seed-based *d* Mapping, a coordinate-based meta-analytic approach to identify consistent regions of GM anomalies across studies of PSP. Totally, 18 original VBM studies, comprising 284 patients with PSP and 367 healthy controls were included. As compared to healthy controls, patients with PSP demonstrated significant GM reductions in both cortical and subcortical regions, including the frontal motor cortices, medial (including anterior cingulate cortex) and lateral frontal cortices, insula, superior temporal gyrus, striatum (putamen and caudate nucleus), thalamus, midbrain, and anterior cerebellum. Our study further suggests that many confounding factors, such as age, male ratio, motor severity, cognitive impairment severity, and illness duration of PSP patients, and scanner field-strength, could contribute to the heterogeneity of GM alterations in PSP across studies. Our comprehensive meta-analysis demonstrates a specific neuroanatomical pattern of GM atrophy in PSP with the involvement of the cortical-subcortical circuitries that mediate vertical supranuclear gaze palsy, motor disabilities (postural instability with falls and parkinsonism), and cognitive-behavioral disturbances. Confounding factors merit attention in future studies.

## INTRODUCTION

Progressive supranuclear palsy (PSP) is a clinical syndrome characterized mainly by early postural instability with falls, vertical supranuclear gaze palsy, parkinsonism, and cognitive-behavioral disturbances that lead to significant disabilities with a mean survival of 6.38 years [1–5]. PSP is a rapidly progressive neurodegenerative disorder, pathologically confirmed by the accumulation of tau protein and neuropil threads in cortical and subcortical structures [3, 6]. Several clinical subtypes of PSP have been identified, of which the classic Richardson's syndrome (PSP-RS) and the PSP-parkinsonism variant (PSP-P) are the most common [3]. No effective treatments are available for PSP [7]. Its differential diagnosis from other parkinsonian disorders is critical but presents challenges in clinical practice, especially in the early disease stages [3, 8]. Despite impressive advances in understanding its pathophysiology, the reliably validated biomarkers for the ante-mortem diagnosis and the prognosis of PSP have not yet been established [3, 4, 9, 10].

Major improvements in modern magnetic resonance imaging (MRI) techniques increase our ability to identify brain structural alterations *in vivo* that shed light on the neuroanatomical basis of PSP and hold promise for its diagnosis [4, 11–13]. Midbrain atrophy is a hallmark of PSP [12, 13]. Recent evidence from whole-brain voxel-based morphometry (VBM) studies in PSP has additionally demonstrated gray matter (GM) atrophy in a number of brain regions, such as the thalamus, basal ganglia, insula, frontal cortices, temporal cortices, parietal cortices, and cerebellum [13–31]. Compared with conventional MRI investigations that draw regions of interest (ROIs) for morphometric comparisons, VBM is a hypothesis-free analytic tool to quantify regional structural differences between groups at a whole-brain level [32]. VBM has been widely used in neurodegenerative disorders [33–35]. Despite the strengths, inconsistent results across different VBM studies were reported [13]. Shi, et al. in 2012, Shao, et al. in 2013, and Yu, et al. in 2014 thus conducted three coordinate-based meta-analyses to test the consistency of GM changes in PSP, which included nine, nine, and 12 VBM studies, respectively [36–38]. However, these meta-analyses had several limitations. First, these meta-analyses did not examine the confounding variables, such as age, gender, disease duration, and symptom severity that potentially lead to heterogeneity of the structural alterations associated with PSP [13]. Second, the quantitative voxel-based meta-analytic tools have been modified [39–41]. Several complementary analyses, such as jackknife sensitivity, heterogeneity, and publication bias analyses could be further performed to explore the robustness of the findings [42, 43]. Third, the numbers of VBM studies included in these meta-analyses were limited. To achieve sufficient power for moderate effects, Eickhoff and co-workers recently recommended that 17 or more experiments were needed for a coordinate-based meta-analysis to detect moderately sized effects [39]. In recent two years, we identified six more VBM studies of PSP eligibly for the meta-analysis. As such, results from previous meta-analyses cannot be considered conclusive and will need replication in a more exhaustive meta-analysis.

Against this background, we aimed to conduct an updated meta-analysis based on 18 whole-brain VBM studies in PSP using a modified Seed-based *d* Mapping (SDM) approach to obtain more accurate results. In addition, analyses of jackknife sensitivity, heterogeneity, publication bias and meta-regression were comprehensively performed to examine the robustness and replicability of GM abnormalities associated with PSP.

## RESULTS

### Characteristics of included studies

**Figure 1:**
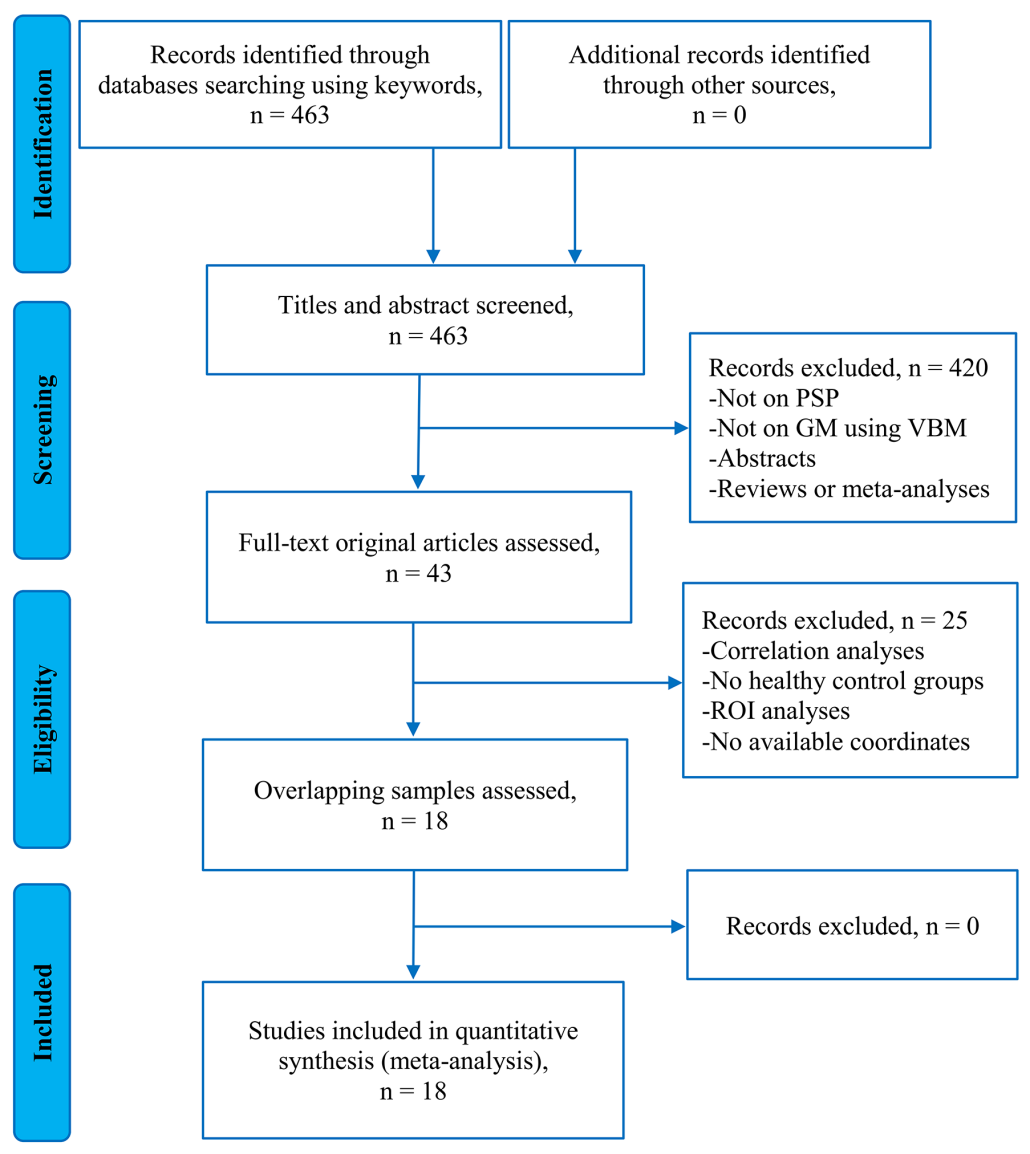
Flowchart to identify the eligible studies for the meta-analysis **Key:** PSP, Progressive Supranuclear Palsy; GM, Gray Matter; VBM, Voxel-Based Morphometry; ROI, Region Of Interest.

Figure 1 presents the flow diagram for inclusion/exclusion of studies in the meta-analysis. Totally, 18 original studies reporting GM differences between 284 patients with PSP and 367 healthy controls were included in this meta-analysis [14–31]. Across the 18 studies, one study included all pathologically confirmed patients with nonfluent/agrammatic variant of primary progressive aphasia (nfvPPA) and PSP (nfvPPA-PSP) [30] and another two studies included some pathologically confirmed patients with PSP in the samples [21, 31]. Patients with PSP in the remaining 15 studies were clinically diagnosed. Three of the 17 studies explicitly indicated that the samples included two subtypes of PSP, PSP-RS and PSP-P [18, 27, 29]. Patients in the remaining 14 of 17 studies were clinically diagnosed based on the NINDS-SPSP clinical criteria [44, 45], which has over 95% sensitivity and specificity in diagnosing PSP-RS [45]. No significant differences between patients with PSP and healthy controls regarding mean age (standardized mean difference = 0.17; 95% confidence interval [CI] = -0.003 to 0.344, z = 0.61, p = 0.055) or gender distribution (relative risk = 1.12, 95% CI = 0.974 to 1.289, z = 0.159, p = 0.112) were observed. Mean Unified Parkinson's Disease Rating Scale-motor examination (UPDRS-III) score in 11/18 studies (rang from 20.4 to 52.9), Hoehn and Yahr disability scale (H&Y) in 7/18 studies (rang from 2.6 to 3.8), illness duration in 15/18 studies (rang from 2.5 years to 4.8 years), Mini-Mental State Examination (MMSE) score in 13/18 studies (rang from 21 to 28), and Frontal Assessment Battery (FAB) examination in 6/18 studies (rang from 7.81 to 12.9) were reported. Nine out of the 18 studies were conducted on 1.5T MRI systems and 8/18 studies were on 3.0T systems. One study used either a 1.5T or a 3.0T MRI system. 13 out of the 18 studies reported the corrected results and the remaining 5 studies used the uncorrected thresholds. All the studies included used Statistical Parametric Mapping (SPM) softwares for imaging analyses.

The quality score of each study included in this meta-analysis was not less than 8 (a maximum score of 10 for each study), which indicates the high rigour of these studies. The list, demographic, clinical and imaging characteristics, and scores of quality assessment of the included studies are presented in Table 1.

**Table 1:** Demographic, clinical and imaging characteristics of VBM studies included in the meta-analysis

Study	Sample (male)	Age (SD)	UPDRS-III (SD)	H&Y stage (SD)	Duration (SD)	MMSE (SD)	FAB (SD)	Scanner	Software	FWHM	Threshold	Quality^#^
**Brenneis et al. (2004)**	PSP 12 (NA)HC 12 (NA)	67.5 (6.6)60 (5.8)	38.9 (10.9)	NA	2.7 (0.9)	NA	NA	1.5T	SPM99	10	p < 0.05corrected	8.5
**Price et al. (2004)**	PSP 12 (7)HC 12 (8)	65.3 (5.8)67.4 (4.6)	20.4 (8.7)	NA	4.8 (1.7)	27 (3.3)	12.4 (3.1)	1.5T	SPM99	8	p < 0.05corrected	9.5
**Cordato et al. (2005)**	PSP 21 (14)HC 23 (14)	70.3 (6.4)71.5 (7.2)	23.1 (10.1)	3.8 (1.1)	4.0 (2.8)	25.4 (3.2)	NA	1.5T	SPM99	12	p < 0.05corrected	9.5
**Boxer et al. (2006)**	PSP 15 (9)HC 80 (37)	70.9 (6.9)67.9 (8.6)	NA	3.3 (0.5)	4.8 (1.7)	24.0 (3.2)	NA	1.5T	SPM2	12	p < 0.05corrected	8.5
**Padovani et al. (2006)**	PSP 14 (7)HC 14 (7)	73 (5.6)65.6 (4.1)	22.1 (8.9)	NA	3.1 (1.0)	25.8 (2.7)	NA	1.5T	SPM2	10	p < 0.005corrected	9.0
**Agosta et al. (2010)**	PSP 20 (14)HC 24 (13)	64.9 (NA)63.8 (NA)	32.8 (NA)	3.0 (NA)	4.5 (NA)	27.0 (NA)	NA	1.5T	SPM5	8	p < 0.001uncorrected	9.0
**Lehericy et al. (2010)**	PSP 10 (6)HC 9 (5)	66.9 (6.4)66.5 (4.8)	30 (NA)	NA	4.3 (1.0)	27 (NA)	11.5 (NA)	1.5T	SPM5	8	p < 0.05corrected	8.5
**Takahashi et al. (2011)**	PSP 16 (11)HC 20 (16)	64.6 (6.4)64.8 (6.4)	NA	NA	NA	21.0 (4.4)	NA	1.5T	SPM8	8	p < 0.001uncorrected	8.5
**Ghosh et al. (2012)**	PSP 23 (14)HC 22 (15)	71.1 (8.6)71.4 (7.6)	33.8 (15.7)	NA	2.5 (NA)	NA	NA	3.0T	SPM5	NA	p < 0.05corrected	9.0
**Giordano et al. (2013)**	PSP 15 (8)HC 15 (8)	68.91 (1.2)65.5 (6.1)	38.33 (4)	3.80 (1.1)	3.16 (1.3)	21.23 (1.2)	7.81 (0.9)	3.0T	SPM8	8	p < 0.05corrected	9.5
**Kamiya et al. (2013)**	PSP 16 (10)HC 21 (12)	71.4 (6.0)70.9 (8.0)	NA	NA	NA	NA	NA	1.5T	SPM5	8	p < 0.001uncorrected	9.0
**Lagarde et al. (2013)**	PSP 19 (7)HC 18 (7)	65.9 (6.5)67.8 (5.2)	NA	NA	4.5 (1.8)	25.5 (2.7)	11.3 (2)	3.0T	SPM8	8	p < 0.05corrected	9.0
**Whitwell et al. (2013)**	PSP 16 (8)HC 20 (4)	72.1 (4.6)73.9 (6.3)	52.9 (12.6)	NA	4.0 (1.1)	25.8 (2.7)	12.9 (2.2)	3.0T	SPM5	8	p < 0.05corrected	9.0
**Sandhya et al. (2014)**	PSP 10 (9)HC 8 (5)	NANA	NA	NA	NA	NA	NA	3.0T	SPM8	NA	p < 0.001uncorrected	8.0
**Burciu et al. (2015)**	PSP 20 (10)HC 20 (10)	67.8 (7.1)64.8 (8.8)	39.0 (14.5)	2.6 (0.9)	2.6 (2.6)	NA	NA	3.0T	SPM8	NA	p < 0.05corrected	9.0
**Piattella et al. (2015)**	PSP 16 (9)HC 16 (6)	68.08 (5.9)69.4 (0.4)	27.0 (17.4)	2.9 (1.0)	3.1 (NA)	24.3 (3.9)	11.1 (3.8)	3.0T	SPM8	12	p < 0.05corrected	9.5
**Wang et al. (2015)**	PSP 24 (8)HC 23 (14)	64.17 (6.72)60.52 (6.47)	NA	3.1 (NA)	3.87 (2.62)	23.54 (4.28)	NA	3.0T	SPM8	6	p < 0.001corrected	8.5
**Santos-Santos et al. (2016)**	PSP 5 (1) HC 10 (3)	71.5 (NA)74 (NA)	NA	NA	4 (NA)	28 (NA)	NA	NA	SPM12	NA	p<0.001uncorrected	8.0

Key: VBM, Voxel-Based Morphometry; PSP, Progressive Supranuclear Palsy; HC, Healthy Controls; UPDRS-III, Unified Parkinson's Disease Rating Scale-motor examination; H&Y, Hoehn and Yahr disability scale; MMSE, Mini-Mental State Examination; FAB, Frontal Assessment Battery; NA, Not Available; SPM, Statistical Parametric Mapping; FWHM, Full Width Half Maximum, SD, Standard Deviation; ^#^, a maximum score of 10 for each study.

### Main voxel-wise meta-analysis

**Figure 2:**
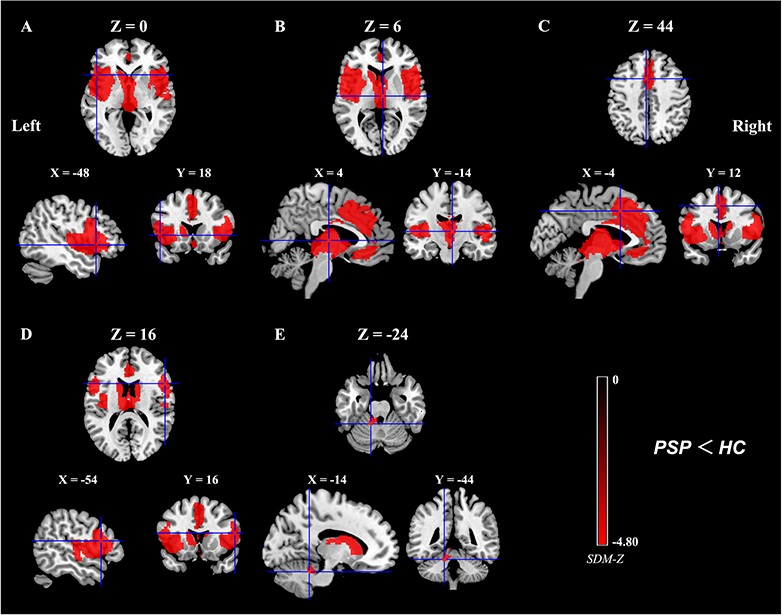
Meta-analytic results of gray matter reductions in patients with PSP compared to healthy controls Key: **(A)**, Left inferior frontal gyrus/insula/superior temporal gyrus/precentral gyrus (premotor cortex)/putamen/ orbitofrontal cortex; **(B)**, Right/Left thalamus/midbrain/caudate nucleus; **(C)**, Right/Left anterior cingulate cortex/(pre-) supplementary motor area/superior medial frontal cortex/medial orbitofrontal cortex; **(D)**, Right inferior frontal gyrus/insula/superior temporal gyrus/putamen/precentral gyrus (premotor cortex); **(E)**, Left anterior cerebellum (lobule III/IV/V); PSP, Progressive Supranuclear Palsy; HC, healthy controls; SDM, Seed-based *d* Mapping. The color bar indicates the maximum and the minimum SDM-Z values.

As shown in Figure 2, the voxel-wise meta-analysis identified significant GM reductions in the left inferior frontal gyrus extending to the insula, superior temporal gyrus, precentral gyrus (premotor cortex), putamen, and orbitofrontal cortex (OFC), in the bilateral thalamus extending to the midbrain and caudate nucleus, in the bilateral anterior cingulate cortex (ACC) extending to the supplementary motor areas (SMA) and pre-SMA, superior medial frontal cortex, and medial OFC, in the right inferior frontal gyrus extending to the insula, superior temporal gyrus, putamen and precentral gyrus (premotor cortex), and in the left anterior cerebellum (lobule III/IV/V) in patients with PSP compared with healthy controls. In contrast, no significant GM increases were observed in patients with PSP relative to healthy controls. Details of the results are summarized in Table 2.

**Table 2:** GM reductions in patients with PSP compared to healthy controls

Cluster	Anatomical label	Peak MNI coordinate (x, y, z)	No. of voxels	SDM-Z value	SDM-p value	Egger's test (p value)
**A**	Left inferior frontal gyrus/insula/superior temporal gyrus/precentral gyrus (premotor cortex)/putamen/OFC (BAs 47, 13, 44, 22, 6, 45, and 9)	-48, 18, 0	5063	-4.80	∼0	0.56
**B**	Right/Left thalamus/midbrain/caudate nucleus	4, -14, 6	3916	-4.70	∼0	0.29
**C**	Right/Left ACC/(pre-) SMA/superior medial frontal cortex/medial OFC (BAs 32, 24, 8, 9, 6,11, and 10)	-4, 12, 44	3457	-3.32	0.000067	0.27
**D**	Right inferior frontal gyrus/insula/superior temporal gyrus/putamen/precentral gyrus (premotor cortex) (BAs 44, 13, 47, 22, 6, 45, and 9)	54, 16, 16	3186	-4.27	0.027743R254	0.78
**E**	Left anterior cerebellum (lobule III/IV/V)	-14, -44, -24	108	-2.74	0.0014	0.83

Key: GM, Gray Matter; PSP, Progressive Supranuclear Palsy; MNI, Montreal Neurological Institute; No., Number; SDM, Seed-based *d* Mapping; BA, Brodmann Area; OFC, Orbitofrontal Cortex; ACC, Anterior Cingulate Cortex; SMA, Supplementary Motor Area.

The subgroup analysis of 14 VBM studies that patients were suggestive of PSP-RS showed that the results remained largely unchanged.

### Supplemental analyses

The jackknife sensitivity analysis revealed that regions of GM reductions in left inferior frontal gyrus extending to the insula, superior temporal gyrus, precentral gyrus (premotor cortex), putamen, and OFC, in the bilateral thalami extending to the midbrain and caudate nucleus, in the bilateral ACC extending to the (pre-) SMA, superior medial frontal cortex, and medial OFC, and in the right inferior frontal gyrus extending to the insula, superior temporal gyrus, putamen and precentral gyrus (premotor cortex) in patients with PSP relative to healthy controls were replicable in all 18 studies. The region of GM reductions in the left anterior cerebellum (lobule III/IV/V) in patients with PSP relative to healthy controls was replicable in 15 studies (Table 3).

**Table 3:** Jackknife sensitivity analysis

All studies but …	A	B	C	D	E
**Brenneis et al. (2004)**	Yes	Yes	Yes	Yes	Yes
**Price et al. (2004)**	Yes	Yes	Yes	Yes	Yes
**Cordato et al. (2005)**	Yes	Yes	Yes	Yes	Yes
**Boxer et al. (2006)**	Yes	Yes	Yes	Yes	Yes
**Padovani et al. (2006)**	Yes	Yes	Yes	Yes	Yes
**Agosta et al. (2010)**	Yes	Yes	Yes	Yes	Yes
**Lehericy et al. (2010)**	Yes	Yes	Yes	Yes	Yes
**Takahashi et al. (2011)**	Yes	Yes	Yes	Yes	Yes
**Ghosh et al. (2012)**	Yes	Yes	Yes	Yes	No
**Giordano et al. (2013)**	Yes	Yes	Yes	Yes	Yes
**Kamiya et al. (2013)**	Yes	Yes	Yes	Yes	No
**Lagarde et al. (2013)**	Yes	Yes	Yes	Yes	Yes
**Whitwell et al. (2013)**	Yes	Yes	Yes	Yes	Yes
**Sandhya et al. (2014)**	Yes	Yes	Yes	Yes	Yes
**Burciu et al. (2015)**	Yes	Yes	Yes	Yes	Yes
**Piattella et al. (2015)**	Yes	Yes	Yes	Yes	Yes
**Wang et al. (2015)**	Yes	Yes	Yes	Yes	No
**Santos-Santos et al. (2016)**	Yes	Yes	Yes	Yes	Yes
**Total**	**18 out of 18**	**18 out of 18**	**18 out of 18**	**18 out of 18**	**15 out of 18**

Key: A, Left inferior frontal gyrus/insula/superior temporal gyrus/precentral gyrus (premotor cortex)/putamen/orbitofrontal cortex; B, Right/Left thalamus/midbrain/caudate nucleus; C, Right/Left anterior cingulate cortex/(pre-) supplementary motor area/superior medial frontal cortex/medial orbitofrontal cortex; D, Right inferior frontal gyrus/insula/superior temporal gyrus/putamen/precentral gyrus (premotor cortex); E, Left anterior cerebellum (lobule III/IV/V); Yes, the cluster reported; No, the cluster not reported.

The heterogeneity analysis revealed that there is significant between-study variability of GM differences in patients with PSP relative to healthy controls in the right inferior frontal gyrus extending to the insula and superior temporal gyrus, in the left insula extending to the superior temporal gyrus, in the bilateral ACC extending to the medial OFC, in the bilateral thalamus, in the left inferior frontal gyrus, in the left cerebellum (lobule III), and in the right caudate nucleus (Table 4).

**Table 4:** Regions of GM heterogeneity from the SDM analysis

Anatomical regions	Maximum MNI coordinate	No. of Voxels	SDM-Z	p
**Right inferior frontal gyrus/insula/superior temporal gyrus (BAs 47, 13, 38, 45, and 48)**	46, 14, 2	1523	4.80	0.0000046
**Left insula/superior temporal gyrus (BAs 47, 13, and 48)**	-40, 0, -2	984	4.59	0.0000077
**Right/Left ACC/medial OFC (BAs 32, 10, 11, and 24)**	2, 50, 8	962	4.26	0.000034
**Right/Left thalamus**	2, -18, -8	306	5.17	∼0
**Left inferior frontal gyrus (BAs 45, and 44)**	-50, 20, 22	178	3.49	0.00044
**Left cerebellum (lobule III)**	-8, -34, -18	37	3.19	0.0011
**Right caudate nucleus**	12, -2, 20	11	2.77	0.0030

Key: GM, Gray Matter; SDM, Seed-based *d* Mapping; MNI, Montreal Neurological Institute; No., Number; BA, Brodmann Area; ACC, Anterior Cingulate Cortex; OFC, Orbitofrontal Cortex.

**Figure 3:**
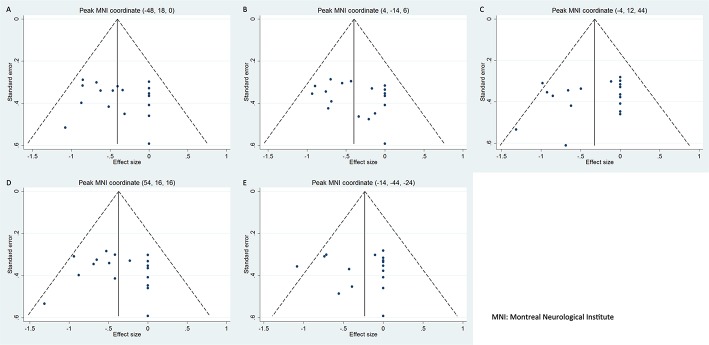
Funnel plots of the peak coordinates of gray matter abnormalities in progressive supranuclear palsy

No publication biases were detected in the regions obtained from the main voxel-wise meta-analysis as revealed by the symmetrical funnel plots (Figure 3) and statistically non-significant Egger's tests (Table 2).

**Figure 4:**
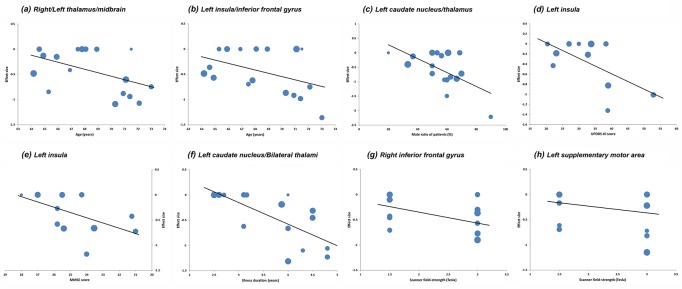
Results of the meta-regression analyses **Key:** UPDRS-III, Unified Parkinson's Disease Rating Scale-motor examination; MMSE, Mini-Mental State Examination. Each study is represented as a dot, with a larger dot indicating a larger sample size. **(A)** and **(B)**, meta-regression with mean age; **(C)**, meta-regression with male ratio of patients; **(D)**, meta-regression with mean UPDRS-III score; **(E)**, meta-regression with mean MMSE score; **(F)**, meta-regression with illness duration; **(G)** and **(H)**, meta-regression with scanner field-strength.

Meta-regression analysis revealed that the PSP group with older mean age (available from 17 studies) exhibited more GM reductions in the bilateral thalamus extending to the midbrain (Figure 4A) and in the left insula extending to the inferior frontal gyrus (Figure 4B). Higher male ratio of patients in the PSP group (available from 17 studies) was associated with more GM reductions in the left caudate nucleus extending to the thalamus (Figure 4C). Higher average UPDRS-III score (Figure 4D) or lower mean MMSE score (Figure 4E) in the PSP group (available from 11 and 13 studies, respectively) correlated with more GM reductions in the left insula. Meta-regression analysis indicated that longer mean illness duration of the PSP group (available from 15 studies) was associated with more GM reductions in the Left caudate nucleus extending to bilateral thalami (Figure 4F). Higher scanner field-strength in VBM studies tended to detect more GM atrophy in the right inferior frontal gyrus (Figure 4G) and in the left SMA (Figure 4H). Findings of these meta-regression analyses are presented in Table 5 and the regression lines are shown in Figure 4.

**Table 5:** Meta-regression analyses

Anatomical label	Peak MNI coordinate (x, y, z)	No. of voxels	SDM-Z value	p value
**Effect of age: GM changes in studies with older patients compared to younger patients**				
**a. Right/Left thalamus/midbrain**	2, -14, 6	655	-4.98	∼0
**b. Left insula/inferior frontal gyrus**	-44, 12, 4	641	-4.15	0.0000026
**Effect of gender: GM changes in studies with a higher male ratio of patients**				
**c. Left caudate nucleus/thalamus**	-6, -4, 14	155	-3.71	0.000014
**Effect of motor severity: GM changes in studies of patients with higher average UPDRS-III score**				
**d. Left insula**	-32, 12, 10	15	-3.38	0.00015
**Effect of cognitive impairment: GM changes in studies of patients with lower average MMSE score**				
**e. Left insula**	-32, 22, 0	41	-3.66	0.00015
**Effect of illness duration: GM changes in studies of patients with longer average illness duration**				
**f. Left caudate nucleus/thalamus/Right thalamus**	0, -8, 12	1447	-6.00	∼0
**Effect of scanner field-strength: GM changes in studies of patients with higher scanner field-strength**				
**g. Right inferior frontal gyrus**	54, 12, 16	127	-4.02	0.000037
**h. Left supplementary motor area**	-2, 24, 56	11	-3.49	0.00032

Key: GM, Gray Matter; MNI, Montreal Neurological Institute; No., Number; SDM, Seed-based *d* Mapping; BA, Brodmann Area; UPDRS-III, Unified Parkinson's Disease Rating Scale-motor examination; MMSE, Mini-Mental State Examination.

## DISCUSSION

Using a modified SDM approach, the present quantitative meta-analysis is timely given with a sufficient number of VBM studies that have recently become available. This comprehensive study synthesized the findings from 18 VBM studies comprising 284 patients with PSP and 367 healthy controls. As compared to healthy controls, patients with PSP demonstrated significant GM reductions in both cortical and subcortical regions, including the inferior frontal gyrus extending to the insula, superior temporal gyrus, putamen, precentral gyrus (premotor cortex) and OFC, the thalamus extending to the midbrain and caudate nucleus, the ACC extending to the (pre-) SMA, superior medial frontal cortex, and medial OFC, and the anterior cerebellum (lobule III/IV/V). These GM changes in PSP were highly robust as verified by jackknife sensitivity analyses. In addition, no publication biases in these regions were observed. However, the heterogeneity analysis revealed a significant between-study variability of GM atrophy differences in some of these regions. Further meta-regression analyses indicated that these variations in GM alterations across VBM studies were correlated with the mean age, male ratio, UPDRS-III score, MMSE score and illness duration of PSP patients, as well as scanner field-strength employed.

The pattern of GM atrophy in PSP identified in our meta-analysis is consistent with the histopathological distribution of neuronal loss, gliosis, and accumulation of tau proteins in the midbrain, diencephalon, basal ganglia, cerebellum, frontal and temporal cortices [46]. Midbrain atrophy, which is consistently validated by many imaging modalities, is the most characteristic alteration of PSP [47]. The hallmark of the disease, vertical supranuclear gaze palsy, is considered to correlate with the neurodegeneration of the rostral interstitial nucleus of the medial longitudinal fasciculus (riMLF and the interstitial nucleus of Cajal located in the midbrain, and the central mesencephalic reticular formation [48–50]. In addition, a recent study by Amtage and colleagues employing 18F-Fluorodeoxyglucose (FDG) positron-emission tomography (PET) suggests that the ACC (cingulate eye field), which connections the supplementary eye field, frontal eye field and midbrain regions [51], plays an important role in downward gaze palsy in PSP [52]. The substantia nigra in the midbrain, coupled with the basal ganglia, thalamus, motor cortices, and anterior cerebellum are hubs of a motor control network [53–55]. GM atrophy in these brain regions identified in the current meta-analysis, probably indicative for damage of this network, contributes to the pathophysiology of parkinsonism, such as rigidity, bradykinesia, and postural instability in patients with PSP [3, 55, 56]. Recent evidence suggests that gait disturbance with early falls, one of the characteristic clinical features of PSP, are closely associated with the thalamic dysfunction, which influences the mesencephalic brainstem-thalamus loop [56].

Beyond the motor control, the subcortical structures such as the the substantia nigra, basal ganglia and thalamus are also implicated in mediating cognition and behavior via the frontal-subcortical circuits [57–59]. In addition to the motor symptoms, patients with PSP are frequently accompanied by cognitive-behavioral disturbances, such as executive dysfunction, apathy, and disinhibition, which are prevalent and may occur early in the disease course affecting their quality of daily life [57, 60]. Early cognitive impairment in PSP is shown to be an independent predictor of shorter survival [61–63]. PSP is typically considered a “subcortical dementia” with the impairment of the frontal-subcortical circuits, prominently attributed to the subcortical pathology [59, 63, 64]. Resting-state functional MRI studies have demonstrated a widespread disruption of cortical-subcortical connectivity involved in cognitive and motor dysfunction in PSP [65–67]. Previous studies using manual ROI approaches for the frontal lobe, demonstrated that the severity of behavioral and cognitive disturbances was associated with the degree of frontal atrophy in PSP patients [68–70]. In addition, a longitudinal ROI study further showed that the progression of executive dysfunction correlated with increased rates of frontal atrophy in patients with PSP [71]. A VBM study demonstrated that the severity of behavioral disturbances in mid-stage PSP correlated with atrophy of the OFC surrounding the inferior frontal sulcus and the midbrain [31]. In accordance with these data, our voxel-wise meta-analysis identified frontal GM atrophy noted in the lateral (inferior frontal cortex extending to OFC) and medial (ACC extending to superior medial frontal cortex and medial OFC) frontal cortices, apart from the frontal motor associated cortices including the (pre-) SMA and the premotor cortex. In addition, we identified extra-frontal GM atrophy in the insular cortex and superior temporal cortex. The insula has rich connections with the frontal and subcortical structures acting as a hub for integrating cognitive-affective, sensorimotor, and autonomic information [72, 73]. Our meta-regression analyses showed that the severity of the motor disabilities and cognitive impairment as well as the illness duration of PSP had notable effects on GM atrophy in the insula. Atrophy of the superior temporal cortex along with the OFC, parts of the OFC-subcortical circuit may be associated with disinhibition, one of the frequently observed behavioral symptoms in PSP [58–60, 74]. The ACC is engaged in the medial frontal-subcortical circuit and in the dorsolateral prefrontal-subcortical circuit, dysfunction of which are responsible for apathy and executive dysfunction, respectively [57–59, 74, 75]. In contrast to previous meta-analyses [33–35], cortical atrophy in the current study was more prominent, which may be attributed to the methodological improvement and sufficient statistical power with enough studies as discussed in the introduction [39–41]. Taken together, GM matter atrophy in these cortical and subcortical regions identified in the meta-analysis may shed light on the pathophysiology of the cognitive-behavioral disturbances in PSP.

Notably in the current meta-analysis, we observed heterogeneity of brain GM alterations in some of the regions across studies, which are attributed to the confounding factors, such as age, male ratio, motor severity, MMSE score, and illness duration of PSP patients, and scanner field-strength that were not analyzed by previous meta-analysis [36–38]. For example, meta-regression analysis revealed that older mean age in PSP patients was associated with more GM atrophy in the bilateral thalamus extending to the midbrain and the left insula extending to the inferior frontal gyrus. Age is an important risk factor for PSP [76]. Severer motor disabilities and cognitive-behavioral disturbances in PSP are associated with more GM atrophy in the left insula. The human insula is strongly interconnected with the basal ganglia and cortical regions, which is implicated in cognitive/affective and sensorimotor processing [77, 78]. These brain structure-behavior correlations provided additional insight into the neurobiology of PSP. The epidemiologic data shows that PSP affects men more frequently than women [76, 79]. In the current meta-analysis, we noted that samples with PSP with a higher male ratio tended to have more GM atrophy in the left caudate nucleus extending to the thalamus, which may provide a neuroanatomical basis of such gender susceptibility. However, we could not find the factors that contribute to the heterogeneity of GM changes in the bilateral ACC extending to the medial OFC. As mentioned above, these regions are implicated in cognitive-behavioral disturbances. Due to the limited data of frontal assessment battery and frontal behavioral inventory available from the original studies, we could not further explore the source of GM heterogeneity in these regions. More studies are warranted to assess cognitive-behavioral disturbances and to conduct clinical-neuroanatomical correlations in PSP.

### Limitations

Some limitations of this meta-analysis warrant consideration. First, an intrinsic limitation for coordinate-based meta-analytic approaches is that they are based on coordinate data rather than raw imaging data, which may bias the results [40, 80]. Second, due to that fact that most of the samples were not pathologically confirmed, the clinically heterogeneous nature of PSP might limit specificity of the findings, although the subgroup analysis indicates that this pattern of GM atrophy is specific for classic PSP-RS patients. Further studies with large homogenous samples both by clinically diagnosed and pathologically confirmed are warranted to validate these findings.

## MATERIALS AND METHODS

### Literature search and selection

As the VBM method was introduced in the year of 2000 [32], we systematically searched PubMed, Embase, and Web of Science databases between January 1, 2000 and September 17, 2016 using the Medical Subject Heading (MeSH) term “progressive supranuclear palsy” and its corresponding free terms, and the keywords “voxel-based morphometry” or “vbm” or “gray matter” or “grey matter” or “voxel*”. Furthermore, we checked the bibliographies of relevant review papers and retrieved articles by hand for additional studies. One study was considered for inclusion in the meta-analysis if it (1) was published in an English-language peer-reviewed journal as an original article; (2) reported regional GM changes using a whole-brain VBM analysis for direct comparison between patients with PSP and healthy controls; (3) reported three-dimensional coordinates of maxima (x, y, z) in a standardized stereotaxic space (i.e., Montreal Neurological Institute [MNI] or Talairach); (4) reported significant results of regional GM differences within one study using a constant threshold. Only the baseline dataset was included if the study was longitudinal. Studies were excluded if they limited their analyses to specific regions of interest (ROIs) or volume of interest (VOI). A study was excluded if its sample overlapped with another publication. The quality of each study included in this meta-analysis was evaluated using a 10point checklist that integrated both the clinical and demographic information and the imaging-specific methodology (Supplementary Table 1), which was based on previous meta-analytic studies [81, 82]. Recorded data were extracted from original studies, including the first author's name, year of publication, age, gender and number of patients and controls, clinical variables (e.g., illness duration, UPDRS-III score, H&Y stage, MMSE score, and FAB score), and the imaging characteristics (e.g., scanner field-strength, processing software, full width half maximum [FWHM] and statistical threshold). In addition, peak coordinates and effect sizes (e.g., t-values) of GM differences between patients with PSP and healthy controls from each VBM study were extracted for the following voxel-wise meta-analysis. Two investigators independently performed literature search and selection, assessment of study quality, and data extraction. Any discrepancies were discussed with another investigator until they were resolved. This study followed the Meta-analysis Of Observational Studies in Epidemiology (MOOSE) guidelines [83].

### Data analysis

### Main voxel-wise meta-analysis

Voxel-wise meta-analysis of regional GM differences between patient with PSP and healthy controls was conducted using the modified SDM software package available at http://www.sdmproject.com. The details of the approach have been described in other publications [40, 80, 84–86] and the tutorial available at http://www.sdmproject.com/software/tutorial.pdf An effect-size signed map and an effect-size variance map of the GM differences was first separately recreated for each study. The mean map was then created by voxel-wise calculation of the random-effects mean of the study maps, which was weighted by the sample size, intra- study variability, and additional between-study heterogeneity. Statistical significance was set at a default un-normalised Gaussian kernel kernel size and threshold (FWHM = 20 mm, p = 0.005, peak height Z = 1, cluster extent = 10 voxels), which provided the optimal balance of false positives and negatives [40, 80]. It must be noted that this un-normalised kernel is not designed to smooth any image but to assign indicators of proximity to reported coordinates [80, 84].

In addition, we conduct a subgroup analysis of VBM studies that patients met the NINDS-SPSP (NINDS-SPSP, National Institute of Neurological Disorders and Stroke and Society for Progressive Supranuclear Palsy) criteria suggestive of PSP-RS [44, 45].

### Supplemental analyses

A leave-one-out and whole-brain voxel-based jackknife analysis was performed to assess the sensitivity of the results by iteratively repeating the same analysis, discarding one study each time [80, 84].

A heterogeneity analysis was carried out using a random effects model with Q statistics in order to explore which brain regions are more heterogeneous between studies. Jackknife and heterogeneity analyses were thresholded with the same default settings (FWHM = 20 mm, p = 0.005, peak height Z = 1, cluster extent = 10 voxels) [40, 80].

In addition, the Stata/SE 12.0 software (Stata Corp LP, College Station, TX, USA) was used to examine possible publication bias. Funnel plots and Egger's test was performed by extracting the values from the meta-analytic peaks [42]. An asymmetry of funnel plots and a p-value less than 0.05 of Egger's test were considered significant.

Meta-regression analyses were further conducted to explore the effects of age, gender, UPDRS-III score, MMSE score, illness duration, and scanner field-strength that could potentially influence the meta-analytic results. Statistical significance was thresholded at a more conservative p-value less than 0.0005 and cluster extent more than 10 voxels [80, 85]. Variables, such as H&Y stage, FAB, frontal behavioral inventory (FBI), and the Progressive Supranuclear Palsy Rating Scale (PSPRS), could not be explored by meta-regression analyses due to limited information that was available from less than 10 original studies.

## CONCLUSIONS

In summary, our comprehensive meta-analysis demonstrates a specific pattern of GM atrophy in PSP with the involvement of the cortical-subcortical circuitries in the pathophysiology of the supranuclear gaze palsy, motor disabilities, and cognitive-behavioral disturbances. These morphological findings may have implications for neuroanatomical diagnostic biomarkers of PSP. In addition, our study indicates that many confounding factors contribute to the heterogeneity of GM alterations in PSP across studies, which merits much attention in further studies.

## SUPPLEMENTARY MATERIALS TABLE



## Abbreviations

VBM: voxel-based morphometry
PSP: progressive supranuclear palsy
GM: gray matter
PSP-RS: PSP Richardson's syndrome
PSP-P: PSP-parkinsonism variant
MRI: magnetic resonance imaging
ROIs: regions of interest
SDM: Seed-based *d* Mapping
nfvPPA-PSP: nonfluent/agrammatic variant of primary progressive aphasia and PSP
MeSH: Medical Subject Heading
MNI: Montreal Neurological Institute [MNI]
VOI: volume of interest
UPDRS-III: Unified Parkinson's Disease Rating Scale-motor examination
H&Y: Hoehn and Yahr
MMSE: Mini-Mental State Examination
FAB: frontal assessment battery
FWHM: full width half maximum
MOOSE: Meta-analysis Of Observational Studies in Epidemiology
NINDS-SPSP: National Institute of Neurological Disorders and Stroke and Society for Progressive Supranuclear Palsy
PSPRS: Progressive Supranuclear Palsy Rating Scale
CI: confidence interval
OFC: orbitofrontal cortex
ACC: anterior cingulate cortex
SMA: supplementary motor areas
BA: Brodmann Area
