# Supplementary material for: Brain gray matter abnormalities in progressive supranuclear palsy revisited
Source: Oncotarget. 2017 Sep 15;8(46):80941–55. doi: 10.18632/oncotarget.20895 (PMC5655252; doi:10.18632/oncotarget.20895)
Supplement: Supplementary file 1 [file oncotarget-08-80941-s001.pdf]

# Brain gray matter abnormalities in progressive supranuclear palsy revisited

## SUPPLEMENTARY MATERIALS

**Supplementary Table 1: Quality evaluation checklists (score 0/0.5/1 per item; total score out of 10)***

| |
|---|
| **Category 1: Participants** |
| 1. Patients were evaluated prospectively, certain diagnostic criteria were used, and demographic characteristics were reported. |
| 2. Healthy controls were evaluated prospectively, psychiatric and medical diseases were excluded. |
| 3. Essential variables (e.g., age, gender, illness duration, symptom severity) were checked either by stratification or statistics. |
| 4. Both male and female participants were included and sample size in each group > 10. |
| **Category 2: Methodology for image acquisition and process** |
| 5. Whole-brain level analysis was automated with no priori selection of regions. |
| 6. Spatial coordinates were reported in a standard space (e.g., Talairach or MNI coordinates) |
| 7. The imaging techniques utilized were clearly described for reproducibility. |
| 8. Measurements were clearly described for reproducibility. |
| **Category 3: Results and conclusions** |
| 9. Statistical parameters for both significant and critical non-significant differences were reported. |
| 10. Conclusions were consistent with the results and the limitations were discussed. |
| *For criteria partially met, 0.5 points were given. |

**Key:** MNI, Montreal Neurological Institute.
